# Tolerance of Sponge Assemblages to Temperature Anomalies: Resilience and Proliferation of Sponges following the 1997–8 El-Niño Southern Oscillation

**DOI:** 10.1371/journal.pone.0076441

**Published:** 2013-10-07

**Authors:** Francisco Kelmo, James J. Bell, Martin J. Attrill

**Affiliations:** 1 Instituto de Biologia, Universidade Federal da Bahia, Campus Universitário de Ondina, Salvador, Bahia, Brazil; 2 School of Biological Sciences, Victoria University of Wellington, Wellington, New Zealand; 3 Marine Institute, Plymouth University, Drake Circus, Plymouth, United Kingdom; University of Genova, Italy

## Abstract

Coral reefs across the world are under threat from a range of stressors, and while there has been considerable focus on the impacts of these stressors on corals, far less is known about their effect on other reef organisms. The 1997–8 El-Niño Southern Oscillation (ENSO) had notable and severe impacts on coral reefs worldwide, but not all reef organisms were negatively impacted by this large-scale event. Here we describe how the sponge fauna at Bahia, Brazil was influenced by the 1997–8 ENSO event. Sponge assemblages from three contrasting reef habitats (reef tops, walls and shallow banks) at four sites were assessed annually from 1995 to 2011. The within-habitat sponge diversity did not vary significantly across the study period; however, there was a significant increase in density in all habitats. Multivariate analyses revealed no significant difference in sponge assemblage composition (ANOSIM) between pre- and post-ENSO years for any of the habitats, suggesting that neither the 1997–8 nor any subsequent smaller ENSO events have had any measurable impact on the reef sponge assemblage. Importantly, this is in marked contrast to the results previously reported for a suite of other taxa (including corals, echinoderms, bryozoans, and ascidians), which all suffered mass mortalities as a result of the ENSO event. Our results suggest that of all reef taxa, sponges have the potential to be resilient to large-scale thermal stress events and we hypothesize that sponges might be less affected by projected increases in sea surface temperature compared to other major groups of reef organisms.

## Introduction

Coral reefs around the world are under threat from a range of local- and global-scale threats [1], [2], [3]. At local scales, these include habitat destruction, overfishing, pollution, sedimentation and invasive species. While these threats can have devastating impacts on reefs, in most cases management intervention and mitigation is possible at the scale of the impact. However, for global scale threats, particularly those related to climate change, ocean acidification and climatic variation (e.g. El-Niño events), mitigating and managing these impacts is much more challenging. While the consequences of these larger scale impacts on corals have received considerable attention, far less is known about their likely impacts on other non-calcifying reefs organisms (see [4]). For example, while the effects of large-scale El-Niño Southern Oscillation (hereafter ENSO) events on coral communities have been well described, particularly as a result of the 1997–8 event that had devastating impacts of many coral reefs ([5], [6] many others), the influences of ENSO events on other dominant reef organisms are much less well known.

Sponges are a major component of coral reef communities across the world (e.g. [7], [8], [9]), with a range of important functional roles, from efficiently processing vast quantities of water and stripping food particles, to acting as a major eroders of the carbonate reef structure [10], [11]). Sponges also form important relationships with a range of microorganisms [12], which can facilitate high levels of benthic primary production and nutrient cycling. Despite the fact that sponges are such important components of reefs, we still understand comparatively little about their ecology and stress responses compared to corals (highlighted by [4], [13]).

Declines in coral abundance have been well-documented world-wide (e.g. [14], [15]), and while there are many reports of increased algal abundance as coral cover and herbivorous fish abundance decline, through so-called ‘phase-shifts’, there is increasing recognition that other non-coral states are possible (see [13], [16,]). Interestingly, in contrast to the reports of long-term declines in coral abundance, increases in sponge abundance have also been documented (e.g. [17]) and there have been further reports of small-scale (km^2^) changes from coral-dominated to sponge dominated states within the Caribbean (e.g. [18], [19], [20]) and Pacific Oceans [21], [22]. Some declines in tropical sponge abundance have also been reported (e.g. [23]). In most of the cases where sponges have increased in abundance, they appear to have been either resistant to the same stress causing the coral declines or taken advantage of newly available space (see [13]).

While there have been extensive studies into the factors influencing the spatial distribution patterns of sponges, such as sedimentation [7], [24], [25], water flow and wave action [26], [27], light [28], substrate type [29], food availability [30], competition [31], and predation [32], [33], [34], far less is known about the factors driving patterns of temporal variation in tropical sponge assemblages (but see [35]). In particular, there is a paucity of information concerning the impacts of large-scale climate events on sponges (but see [36]) or how future climate change is likely to influence sponge assemblages despite suggestions that this group may be potential ‘winners’ under future climate change scenarios [13], [37], [38].

The 1997–8 ENSO event had a major impact on coral reefs across the world [5], [39], [40], and the northern Bahia coral reefs where no exception. Most invertebrate taxa were severely impacted and experienced mass mortalities: this included corals and other cnidarians [41]; echinoderms [42], [43]; bryozoans [44]; and ascidians [45], attributed to increased Sea Surface Temperature (SST). In Brazil, our understanding of sponge biodiversity has increased significantly over the last two decades, although the majority of the sponge studies in this region are of a taxonomic nature, and so little is known about sponge ecology in this region (see [46], [47], [48], [49], [50] for example) and how these organisms respond to major environmental perturbations. In this paper we address this lack of information by presenting the results of a 17-year study examining changes in sponge assemblages from three different reef habitat types at four different locations spanning the 1997–8 ENSO event (and later smaller ENSO events) in order to assess assemblage-level impact and recovery patterns.

## Materials and Methods

### Study Area

**Figure 1:**
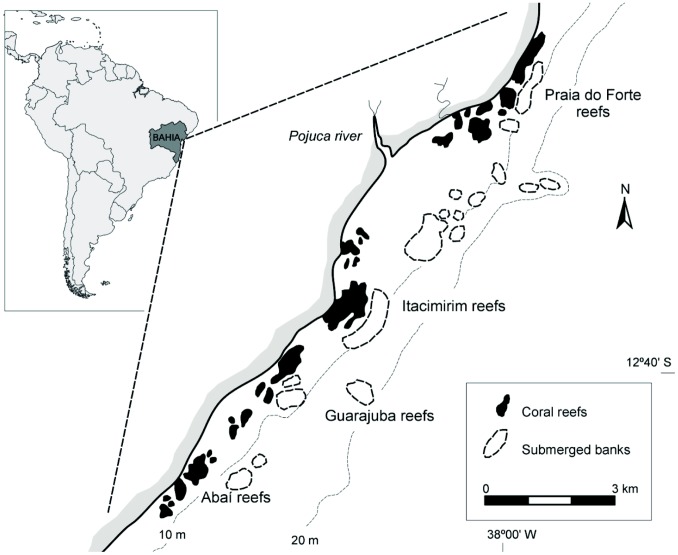
The location of the coral reefs of northern Bahia (After Leão *et al*., 1997).

This study focused on four reefs in Bahia, Brazil [Abaí (12°40′04′′S/38°04′47′′W), Guarajuba (12°39′22′′S/38°03′18′′W), Itacimirim (12°37′20′′S/38°01′40′′W) and Praia do Forte (12°34′42′′S/37°58′59′′W)]. The reefs are on the narrowest part of the Eastern Brazilian Continental Shelf (average width 15 km between the São Francisco and Doce Rivers) and extend 20 km between the beaches of Abai and Praia do Forte (Fig. 1). The studied reefs are all complex elongated structures varying from 500 to 1,800 m in length, 400 to 500 m in width and occur in water depths between 10 and 40 m. The reefs have developed either on rocky outcrops of various ages or on lines of Holocene beachrock [51]. They have an irregular lateral contour, sometimes presenting well-developed, spur-and-groove systems on the fore-reef side, while the back-reef is usually more regular. All four reefs are very similar in terms of morphology and overall species composition (see [52]). Each reef system has three distinct habitats: emergent reef tops, coastal reef walls and offshore shallow bank reefs, all of which were sampled in this study. The emergent reef tops (hereafter ERT) have been eroded due to sea-level fluctuations and have irregular thin columnar structures, cavities, meandering channels, and small caves. Their subtidal edge ranges from 5 to 14 m in depth and represents the coastal reef wall (hereafter CRW) habitat, which ranges in form from near vertical drops to shallower rocky steps. Offshore from each coastal system are a series of shallow bank reefs (hereafter SBR), which are elongated structures that are physically separated from the coastal reef systems and located within a depth range of 10 to 40 m. Leão *et al*. [53] provide a full description of the geological history and morphology of the reefs.

The coastal belt of the State of Bahia has a tropical humid climate. Annual average rainfall ranges between 1,300 mm in the north of the study area to 1,900 mm around Salvador City to the south, with no marked seasonal rainfall pattern. Average daily air temperatures range from 23°C (winter) to 28°C (summer), with mean daily sea-surface temperatures ranging from 25°C (winter) to 28°C (summer); the maximum SST occurs between December and February each year. Annual average salinity varies little (35–36), although within reef-top shallow pools, salinity can range from 35 to 39 (see [52]). The pH of seawater varies only between 8.1 and 8.2, with no clear seasonal patterns (see [54], [55]). The coast is influenced by winds arising from the NE and E during the spring-summer, and winds coming from the SE and E during the autumn-winter season. Moreover, during the autumn-winter period, the winds arising from the SSE, associated with the periodic advance of the Atlantic Polar Front, reinforce the trade winds from the SE [56]. This pattern of wind circulation is disrupted by the quasi-cyclic environmental phenomenon known as the El Niño/La Niña, combined as the El Niño Southern Oscillation, with several major climatic perturbations recorded [57], [58].

### Sampling and Identification

Sponge abundance was quantified annually (between April and May each year) in the three contrasting reef environments (ERT, CRW and SBR) on each of four different reefs in northern Bahia from 1995 to 2011: (i) ERTs were sampled during low tide; and (ii) CRWs and SBRs were sampled by snorkeling or scuba diving. Quantitative samples were taken from 35×1 m^2^ positioned haphazardly quadrats, along a transect line parallel to the coastline, on each reef, totaling 140 quadrats per habitat, 420 quadrats per year, and 7140 quadrats over the survey period. Permanent License to collect zoological material (N° 37409-1) was dispatched on the basis of the Normative Instruction N° 154/2007 by the Ministry of the Environment, Chico Mendes Institute of Biodiversity Conservation, Authorisation System and Information on Biodiversity. Through the authentication code N° 78456982, any citizen can check the authenticity or legality of this document, by means of the page the Sisbio/ICMBio on the Internet (www.icmbio.gov.br/sisbio). No other specific permissions were required due to the fact that this was an entirely field based study with all data being recorded on site through the *in-situ* identification and counting of sponges. The location is not privately-owned or protected in any way, as the beaches surveyed are public spaces. We did not remove or damage any of the studied organisms beyond taking small tissue samples from each species during early years to confirm species identity. Otherwise, sponges were just counted (taking only small samples for confirming field identification of any new or uncertain individuals), so our methods represent no threat to the species we assessed and none is, as yet, endangered.

Because accurate quantification of sponge biomass can be a destructive process that potentially interferes with subsequent sampling, and because of the logistical constraints associated with this large sampling effort, we counted all sponges colonies *in situ* as our measure of abundance resulting in density values per m^2^. We were primarily interested in any loss or additions of sponge species within the reefs, so counting discrete colonies provided the most suitable measure to document disappearance or resettlement. Data were collected on sponge color, shape and size in the field and photographs were taken of each species. The identity of each species was originally confirmed through histological examination, based on authoritative keys and texts (e.g. [47], [48], [50] and references therein). Spicule preparations were made by dissociation of small tissue fragments in boiling nitric acid. Thick sections of specimens were observed under a light microscope to observe the skeletal architecture. Where necessary, microscleres were observed using a scanning electronic microscope (SEM) Zeiss (DSM 940A).

### Environmental Data

Large-scale environmental parameters for the survey area (sea surface temperature, solar irradiance, air temperature, rainfall, and cloud cover) were obtained from the Brazilian Meteorological Institute [INMET (http://www.inmet.gov.br)]. INMET data are collected three times a day and the values presented in this paper represent the annual average of these data. Local physicochemical data (seawater temperature, salinity, pH, and turbidity) were recorded at all four reefs (10 replicates/reef giving 40 measurements spread over the sampling period). Temperature, salinity, and pH were recorded using a YSI63 (Yellow Spring Industries) electronic field meter. Turbidity was assessed using a Secchi disk that was deployed from a boat for CRW and SBR environments. From 2001, we recorded turbidity and other local data using a Multiparameter Water Quality Meter (U5210); however, based on the similarity in the results obtained from the different methods we present the same type of measurement throughout the years to ensure consistency.

### Data Analysis

The sponge density data are expressed as mean ± standard error (SE). We performed, for each contrasting reef environment (ERT, CRW and SBR), a non-metric multidimensional scaling (nMDS) using a Bray-Curtis [59] dissimilarity matrix, which was calculated from log (x+1) transformed density data standardized by sample totals and used several random starts in order to achieve the optimum configuration. The results were visualized by an ordination diagram with 95% confidence ellipses around multivariate centroid of samples from each habitat type. We further used permutational multivariate analysis of variance (PERMANOVA) to test the hypothesis of no significant differences in sponge density and richness between reefs (Praia do Forte, Itacimirim, Guarajuba and Abai) and years (before, during and after 1997–8 ENSO event; a priori year groupings). PERMANOVA allows multivariate information to be partitioned according to the full experimental design. It makes no assumptions regarding the distributions of the original variables and all P-values are obtained by permutation. All tests were carried out using the type III sum of squares and 4999 permutations under the reduced model [60], [61]. Given the high number of permutations run, additional Monte Carlo tests were not necessary to reinforce the permutation P-values obtained [62]. Finally, to investigate the relationship between the measured environmental variables (before, during and after 1997–8 ENSO event) and sponge assemblage data the BIOENV routine (Spearman rank correlation method) was used with biological and environmental data collected during each sampling year. All these analyses were performed with the software package PRIMER (version 6.1.6; PRIMER-E, Plymouth, U.K.) and the PERMANOVA+ module (version 1.0.1. PRIMER-E, Plymouth, U.K.).

## Results

**Figure 2:**
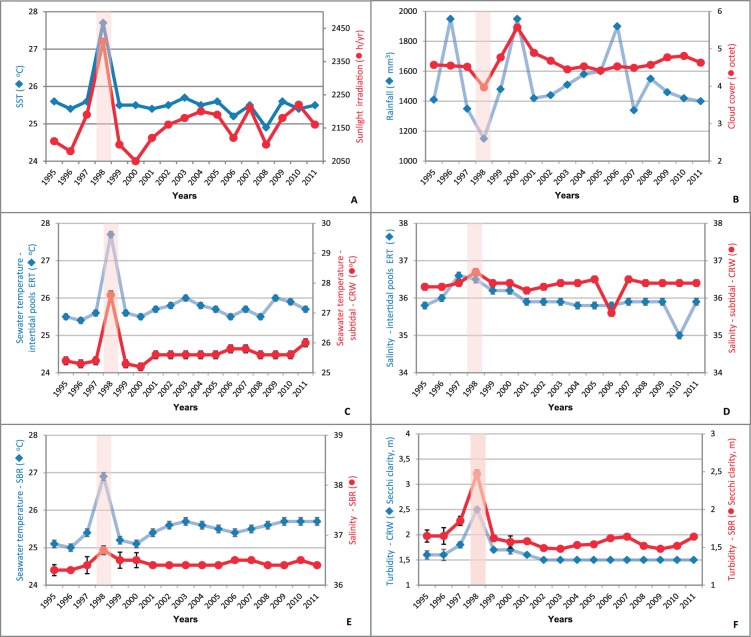
Summary of wide-scale (A–B) and locally measured (C–F) environmental variables recorded from the studied reefs throughout the sampling period, demonstrating changes in ambient conditions during the El Niño period (1998). (A) Annual sea surface temperature and sunlight irradiance; (B) Annual rainfall and mean daily cloud cover; (C) Mean seawater temperature (ERT and CRW); (D) Mean salinity (ERT and CRW); (E) Mean seawater temperature and salinity (SBR); (F) Mean water clarity (CRW and SBR). Error bars indicate SE around the mean between replicate reef systems; Vertical bars represent the timing of the 1997–8 El Niño event.

We found that there were higher seawater temperatures, lower sky cover and lower turbidity during the 1997–8 ENSO period compared to non-ENSO years (Fig. 2), likely resulting in higher levels of UV radiation reaching the reef associated invertebrates in 1998 than in non-ENSO years (or during subsequent weaker ENSO episodes). Although our data indicated slightly lower salinity at ERT habitats during the non-ENSO period, it varied very little at any of the reef habitats during ENSO period. Rainfall was significantly lower during ENSO conditions and this resulted in reduced freshwater and sediment outflow from the local rivers (the mean annual discharge of the São Francisco River was reduced from 32,980 to 1,768 m^3^s^−1^ and that of Doce River from 80.5 to 50.2 m^3^s^−1^) and, thus, significantly clearer water. 1998 was therefore characterized by warmer air and sea temperatures, reduced cloud cover and rainfall, higher incoming solar radiation, and reduced turbidity (due mainly to reduced river runoff following decreased precipitation). Similar, but not so intense, conditions were observed in 2007 and 2010. In contrast, 1999–2000, and to a lesser extent, 1995–6 represented relatively strong La Niña conditions, as indicated by high rainfall and cloud cover (Fig. 2).

**Figure 3:**
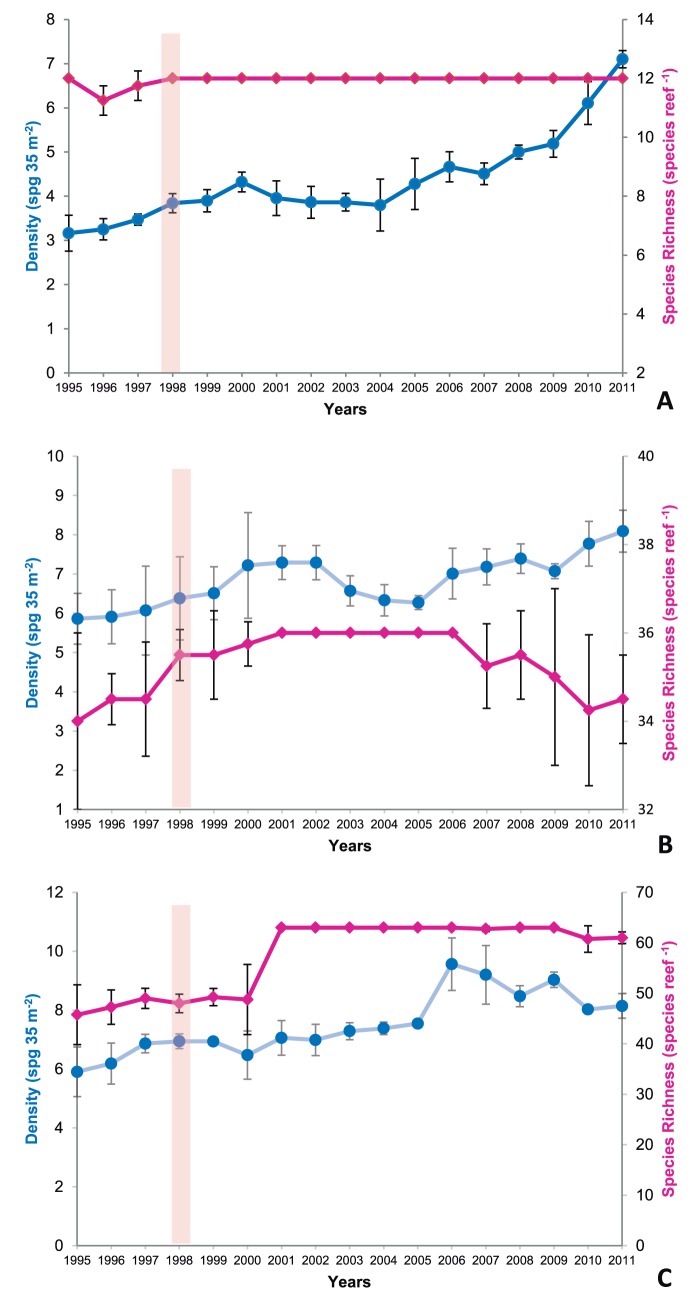
Changes in density and species richness (Mean ± SE) of the sponge assemblage recorded over a 17-year period (1995–2011) in Bahia, Brazil. Vertical bars represent the timing of the severe 1997–8 El Niño.

A total of 63 sponges species (all demosponges; Table S1) were recorded from the three contrasting reefs habitats (ERT: 12, CRW:36, SBR:63) over the sampling period. Overall sponge richness and density increased over the years (Fig. 3). The most abundant species were: *Cinachyrella apion*, *C. alloclada* and the *Cliona celata* complex on the ERT; *Tethya maza, T. rubra, C. apion* and *C. alloclada* on the CRW and *Cliona delitrix* on the SBR.

The PERMANOVA global test results indicated no significant differences in assemblage composition between reefs for both ERT and CRW habitats (Table S2). However, the SBR environment became significantly different throughout the years (increased both density and richness). In addition, we recorded a significant increase in density over time in all three-habitat types since the ENSO event (Fig. 3A–C). The lowest mean sponge density for the ERT assemblages was 3.16±0.2 spg m^−2^, which was recorded in 1995. Density has progressively increased over time on ERTs (Fig. 3A) to a maximum of 7.1±0.09 spg m^−2^ in 2011; a significant overall increase of approximately 100% (pseudo-*F* = 1.9228, P(*perm*) = 0.01). For the CRWs (Fig. 3B), the lowest recorded mean density was 5.86±0.32 spg m^−2^ in 1995, and similar to the ERTs, sponge density has significantly increased throughout the study period to a maximum of 8.09±0.26 spg m^−2^ in 2011 (pseudo-*F* = 2.0023, P(*perm*) = 0.001). The lowest recorded mean sponge density for the SBRs (Fig. 3C) was 5.9±0.42 spg m^−2^ in 1995, which increased significantly to a maximum of 9.5±0.44 spg m^−2^ by 2006 (pseudo-*F* = 2.8913, P(*perm*) = 0.0001), but then decreased slightly to 8.14±0.21 spg m^−2^ in 2011. However, this reduction did not affect the significance of an overall increasing density trend across years. There were no significant changes in sponge species diversity or abundance associated with the 1998 ENSO incident.

**Figure 4:**
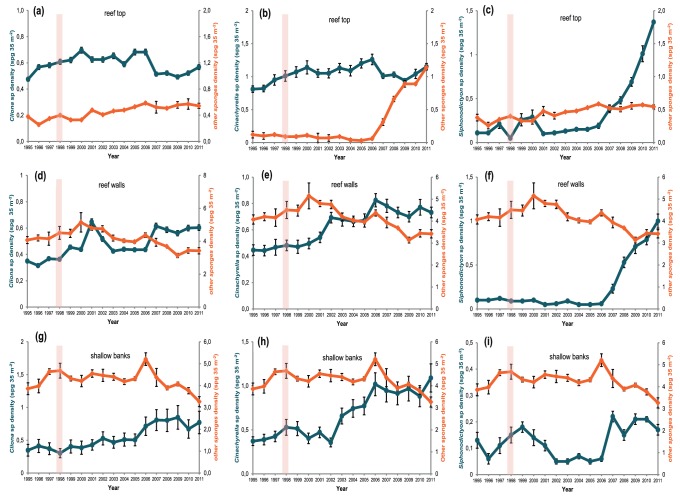
Changes in density of *Cliona* spp., *Cinachyrella* spp. and *Siphonodictyon* spp. recorded from the three contrasting reef habitats from Bahia, Brazil. Vertical bars represent the timing of the 1997–8 El Niño event.

Following the ENSO event, the ERT assemblages (Fig. 4A–C) became progressively dominated by larger populations of three genera: *Cinachyrella* (*C. apion* and *C. alloclada*), *Cliona* [*C. varians* and *C. celata* (complex)] and *Siphonodictyon* (*S. coralliphagum* and *Siphonodictyon* sp.), whose densities had all increased significantly by 2011 compared to 1998. A similar pattern was observed for sponge assemblages at the CRW (Fig. 4D–F) and SBR (Fig. 4G–I) habitats, where the same species became progressively more abundant, although the abundance of some other species decreased including: *Amphimedon viridis*, *Astroclera* sp, *Aplysina cauliformis*, *Callyspongia* (*C. tenerrima* and *C. vaginalis*), *Chondrilla nucula*, *Chondrosia* sp., *Desmapsamma anchorata*, *Dysidea* spp., *Ircinia strobilina*, *Spirastrella cunctatrix* and *Tedania brasiliensis*.

Of particular interest is the significant increase in the densities of two bioeroding genera, *Siphonodictyon* (2 spp.) and *Cliona* (3 spp.). At the beginning of the study, the mean density for both species of *Siphonodictyon* on ERTs was 0.11±0.02 spg m^−2^; however, by 2011 their abundance reached 1.37±0.17 spg m^−2^ [more than ten times their initial density, (pseudo-*F* = 4.6049, P(*perm*) = 0.003)]. A significant increase was also recorded on CRWs with mean densities increasing from 0.1±0.01 in 1995 to 1.0±0.08 spg m^−2^ in 2011 (pseudo-*F* = 82.747, P(*perm*) = 0.0001). This pattern was not observed for SBRs where the mean densities of these species oscillated over time from 0.13±0.03 in 1995 to a maximum of 0.22±0.02 in 2007 and back to 0.17±0.02 spg m^−2^ in 2011, with no significant differences between ENSO and non-ENSO years. *Cliona* spp. density on ERTs increased from 0.48±0.16 in 1995 to 0.7±0.23 spg m^−2^ in 2000, but this value gradually declined to 0.57±0.18 spg m^−2^ in 2011. Significant increases of *Cliona* spp. were recorded at the CRW habitats from 0.35±0.17 in 1995 to 0.6±0.09 spg m^−2^ in 2011(pseudo-*F* = 41.317, P(*perm*) = 0.0001) and in the SBR habitats from 0.35±0.09 to 0.8±0.17 spg m^−2^ over the same period (pseudo-*F* = 14.563, P(*perm*) = 0.0002). However, in contrast to the observations for the coastal reef assemblages, the increase in *Siphonodictyon* density was comparatively much lower than for *Cliona* spp. and the non-bioeroding *Cinachyrella* spp.

**Figure 5:**
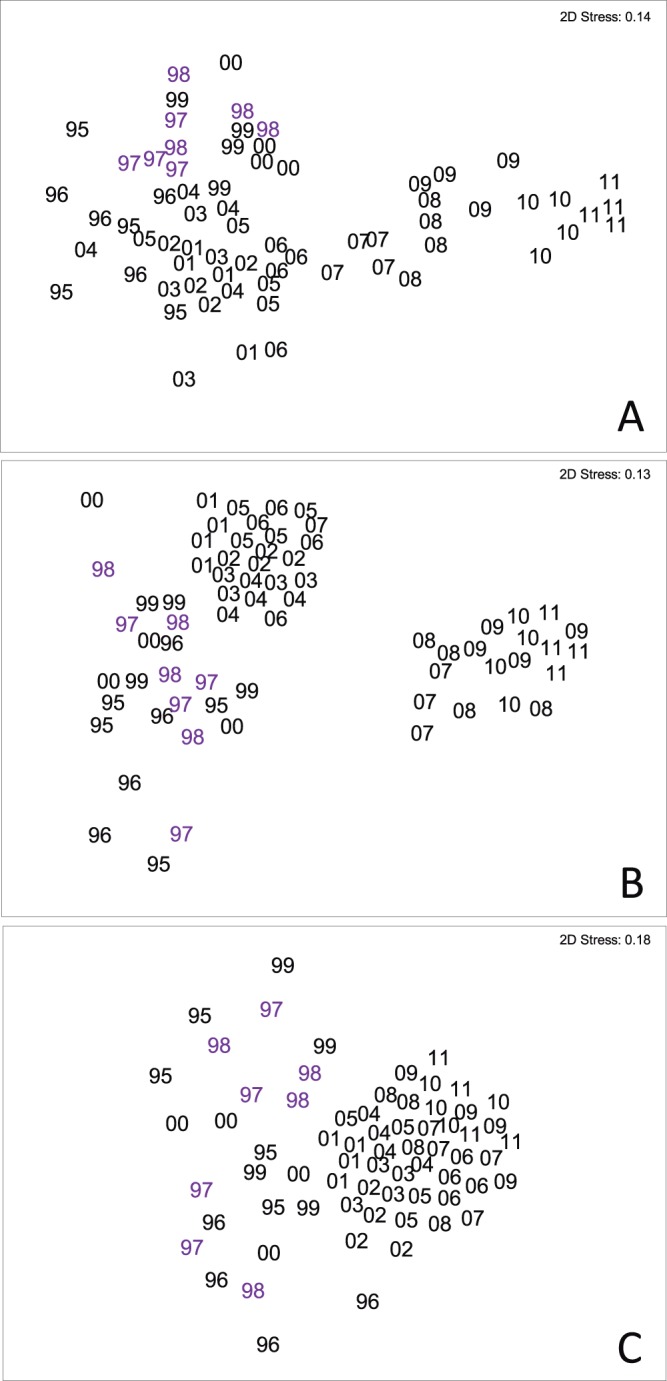
Non-metric multidimensional scaling ordination of sponge assemblage data from the four assessed shallow-bank reefs from northern Bahia (Praia do Forte, Itacimirim, Guarajuba and Abai) throughout the sampling period, 1995–2011. Based on [ln (x+1)]) transformed species densities and Bray Curtis similarities. (A): ERT; (B): CRW; (C): SBR. Codes refer to the year of sampling (i.e. 00 = 2000).

The nMDS ordinations (Fig. 5A–C) for the three contrasting habitats did not show any differences in sponge assemblages between pre-ENSO and ENSO years; however, there were differences in sponge assemblage structure following the ENSO years. For CRWs in particular (Fig. 5B), there was a distinctly different sponge assemblage during the last five years of our study compared to earlier years. In addition, we also found increased richness on the SBR. There was no meaningful correlation with any variables over the time period as sponge assemblages pattern did not follow the main changes associated with ENSO. The highest correlation identified using the BIOENV analysis on ERTs was found for salinity, sunlight irradiation and seawater temperature (r = 0.121), whilst a combination of temperature, sunlight radiation and turbidity best explained the variation in the sponge assemblages on the CRWs (r = 0.131) and SBRs (r = 0.182).

## Discussion

The 1997–8 ENSO had major negative impacts on coral reefs worldwide [63], [64], [65] and for corals the level of recovery from this large-scale event appears to vary considerably between geographic locations [55], [66], [67], [68], [69], [70], [71]. However, far less is known about the longer-term responses of other non-coral organisms to this large-scale climatic event. In this study, we aimed to describe changes in sponge abundance and assemblage composition on a Brazilian coral reef system during this ENSO event and in the subsequent years, and in doing so we present the longest known study of any entire tropical sponge assemblage. We found that sponges appeared unaffected by the increase in seawater temperature and actually increased in abundance after the ENSO event. This is in stark contrast to all other benthic organisms in this study area that experienced mass mortalities, including Foraminifera, corals, echinoderms, bryozoans and ascidians [54], [41], [42], [43], [44], [45]. Our result suggest that tropical sponges in this region may have increased resilience to higher sea surface temperatures compared to other organisms, and this has potentially important implications for reef systems elsewhere.

There are very few long-term studies of entire tropical sponge assemblages, meaning relatively little is known about their patterns of temporal variability (but see [23], [71], [72]), and even less is known about the processes driving such patterns. In one of the longest studies of a sponge assemblage prior to our study, Wulff [23] reported a large decline in a localized Caribbean sponge assemblage in Panama over a 14-year period (1984–1998) in a 16 m^2^ plot. This sponge assemblage lost >50% of sponge species and >40% of biomass over this period, although the decline appeared gradual over time. Interestingly, like the increases in our study, these declines could not be correlated with any specific abiotic or biotic factors, although disease was proposed as a possible cause. This earlier study obviously contrasts with our findings over a similar temporal scale to that of [23] (noting the [23] ended in 1998), where we report an increase in sponge abundance over time, including through a large-scale thermal anomaly event. An important difference between our study and that of Wulff [23] is the different spatial scales that were sampled. We examined sponge assemblages over a much large spatial scale and across a range of different habitat types, whereas the small study area examined by Wulff [23] may have detected only very local-scale effects. We propose the increase in sponges on northern Bahian reefs, at least initially, was the result of increased space availability and potentially reduced competitive interactions as a result of the decline in other benthic groups.

There is increasing evidence that sponges may be potential ‘winners’ in light of global climate change and ocean acidification (see [13] for review). For example, [38] reported that the growth and survival of six ecologically important Caribbean sponge species was similar between treatments consistent with present day conditions (28°C; pH = 8.1) and those predicted for 2100 (31°C; pH = 7.8). However, despite these recent studies there have been earlier reports of negative effects of temperature on sponges (e.g. [73]) and temperature has been implicated in the decline of a number of sponge populations (e.g. [74]). Despite these contrasting results, our study supports the theory that sponges may be more tolerant to climate-associated temperature effects than other benthic groups and thus we suggest that identifying the potential mechanisms enabling sponges to deal with heat stress should be a focus of future investigations.

In addition to ENSO events affecting temperature and light penetration, they also indirectly influence primary production and generally reduce phytoplankton abundance [75]. Given that sponges are suspension feeders, it would be reasonable to suggest that any decline in plankton production would disrupt sponge feeding resulting in decline of the sponge populations during ENSO-events; however, this was not what we observed. Instead, there was an increase in density for both coastal and shallow banks assemblages (Fig. 3) and therefore it appears that demosponges (unlike corals [55]) were unaffected by both the food and temperature stress resulting from the 1997–8 ENSO, or that this perceived feeding disruption does not occur. This might be the result of their ability to feed on organic particle sizes not readily collected by other organisms, particularly pico-plankton [76], [77] or because of the decline of other organisms potentially competing for food (particularly ascidians). Interestingly, the results from a previous study undertaken between 1993–4 at Todos os Santos Bay (approximately 120 km south the studied reefs [78]) provides further data to support sponge assemblages in this region being resilient to stress, as they appeared little affected by the oil pollution that had negative impacts on other organisms.

There have been a number of reports from the Caribbean of increases in bioeroding clionid sponges after coral declines (e.g. [18], [19], [20], [79]); for example [80] reported an increase in *Cliona caribbea* in uncovered coral colonies following the massive bleaching event on the Caribbean coast of Costa Rica during the 1982–3 ENSO event. In our study, densities of *Cliona* actually declined on the SBR during the ENSO period, but they increased significantly in the following years. It is possible that this initial decline was because this genus, like corals, also harbor zooxanthellae [81] and therefore suffered bleaching. Increased abundance by *Cliona* spp. has also been attributed to high organic matter and bacterial loading on reefs [82], [83], [84]. With the exception of *Mycale* sp. and *Cynachirella* (2 spp.), all the other genera recorded from the ERTs were either encrusting or boring species. Amongst them, clionids were abundant and their densities significantly increased over the 17-year investigation (ERT:12%; CRW:17% and SBR:23%), becoming one of the most abundant genera in the SBRs. We believe this is of concern for the long-term stability of this reef system given how they can have a destructive effect on calcium carbonate reef structure.

Anthropogenic impacts have the potential to disrupt the balance between reef erosion and accretion. Cebrian & Uriz [85] observed positive correlations between abundance of *Cliona viridis* and grazing urchin abundance, and a negative correlation with fleshy algae abundance in western Mediterranean. These authors suggested that increased light penetration to the reef as result of algal grazing enhances the growth rate of this sponge species, accounting for its greater abundance in absence of fleshy algae. While it is likely that elevated clionid abundance at our study sites is largely the result of increase substrate availability, urchin numbers were also significantly higher post-ENSO [43] and therefore may have potentially facilitated an increase in clionid sponges. We consider that the major environmental changes associated with the 1997–8 ENSO event provide the most parsimonious explanation for this space liberation and subsequent bioeroding sponge increase as we have no evidence of any other major change in pressure within the reef region, such as fishing intensity, nutrient loading or chemical pollution input.

Even though the overall species richness did not change significantly over time, we identified temporal species-specific changes in abundance, with some species increasing in abundance while others decreased. We propose that these changes are likely the result of biotic, rather than abiotic, interactions because: (i) the environmental conditions during ENSO periods appeared insufficient to notably disturb the sponge assemblage directly; and (ii) because we could not correlate changes in overall abundance with any of the environmental parameters. In particular, we propose that sponges benefited from reduced spatial competition with other benthic groups and increased space availability.

In summary, we found most sponges to be highly resilient to the temperature and UV stress associated with the 1997–8 ENSO event, in contrast to all other benthic groups, which suffered massive mortalities. Furthermore, since this event, sponge abundance has increased, although species richness has remained the same. We propose that, based on the resilience of sponges in this study coupled with results from recent studies (e.g. [13], [37], [38]), sponges may be one benthic group that might withstand the effects of global climate change and actually benefit from the declines expected in other benthic groups.

## Supporting Information

Table S1 Quantitative inventory of accumulated densities (140 ^m-2^) of the Bahian sponge assemblage from the three contrasting reef habitats (ERT; CRW; SBR) of the four reefs assessed throughout the sampling period, 1995–2011.
(DOCX)

Table S2 Statistical differences in sponge assemblages between Reefs/Years measured from 1995 to 2011 tested by a distance-based permutational multivariate analysis of variance, PERMANOVA.
(DOCX)
