# Supplementary material for: Tolerance of Sponge Assemblages to Temperature Anomalies: Resilience and Proliferation of Sponges following the 1997–8 El-Niño Southern Oscillation
Source: PLoS One. 2013 Oct 7;8(10):e76441. doi: 10.1371/journal.pone.0076441 (PMC3792017; doi:10.1371/journal.pone.0076441)
Supplement: Table S1 — Quantitative inventory of accumulated densities (140 m-2) of the Bahian sponge assemblage from the three contrasting reef habitats (ERT; CRW; SBR) of the four reefs assessed throughout the sampling period, 1995–2011. (DOCX) [file pone.0076441.s001.docx]

**Table S1**

Quantitative inventory of accumulated densities (140 m^-2^) of the Bahian sponge assemblage from the three contrasting reef habitats (reef top, reef walls and shallow bank reefs) of the four reefs assessed throughout the sampling period, 1995–2011

| Species | | **1995** | **1996** | **1997** | **1998** | **1999** | **2000** | **2001** | **2002** | **2003** | **2004** | **2005** | **2006** | **2007** | **2008** | **2009** | **2010** | **2011** |
| --- | --- | --- | --- | --- | --- | --- | --- | --- | --- | --- | --- | --- | --- | --- | --- | --- | --- | --- |
| **REEF TOP** | |  |  |  |  |  |  |  |  |  |  |  |  |  |  |  |  |  |
| *Amphimedon viridis* Duchassaing & Michelotti, 1864 | | 0,26 | 0,14 | 0,11 | 0,17 | 0,14 | 0,11 | 0,26 | 0,26 | 0,23 | 0,26 | 0,26 | 0,20 | 0,34 | 0,43 | 0,34 | 0,37 | 0,31 |
| *Chondrilla nucula* (Schmidt, 1862) | | 0,34 | 0,23 | 0,37 | 0,31 | 0,31 | 0,29 | 0,31 | 0,34 | 0,40 | 0,31 | 0,43 | 0,49 | 0,34 | 0,34 | 0,31 | 0,34 | 0,34 |
| *Cinachyrella alloclada* (Uliczka, 1929) | | 3,49 | 3,34 | 3,00 | 3,37 | 3,49 | 4,26 | 4,37 | 4,17 | 3,17 | 3,23 | 3,94 | 4,40 | 4,54 | 5,49 | 5,06 | 5,26 | 5,77 |
| *Cinachyrella apion* (Uliczka, 1929) | | 2,97 | 3,23 | 3,17 | 3,34 | 3,74 | 3,86 | 3,77 | 3,80 | 4,14 | 4,14 | 4,37 | 4,97 | 4,29 | 4,46 | 4,00 | 4,60 | 4,97 |
| *Cliona celata* (Grant, 1826) | | 3,54 | 4,23 | 4,46 | 4,71 | 4,80 | 5,26 | 4,66 | 4,60 | 4,91 | 4,54 | 5,20 | 5,09 | 3,77 | 3,74 | 3,54 | 3,74 | 4,23 |
| *Cliona varians* (Duchassaing & Michelotti, 1864) | | 0,26 | 0,31 | 0,20 | 0,14 | 0,17 | 0,31 | 0,34 | 0,40 | 0,31 | 0,17 | 0,26 | 0,37 | 0,34 | 0,43 | 0,40 | 0,43 | 0,31 |
| *Desmapsamma anchorata* (Carter, 1882) | | 0,23 | 0,20 | 0,26 | 0,17 | 0,26 | 0,20 | 0,43 | 0,31 | 0,37 | 0,26 | 0,40 | 0,43 | 0,37 | 0,26 | 0,34 | 0,37 | 0,31 |
| *Mycale sp1* | | 0,11 | 0,09 | 0,17 | 0,23 | 0,14 | 0,17 | 0,23 | 0,20 | 0,31 | 0,34 | 0,37 | 0,43 | 0,34 | 0,31 | 0,29 | 0,37 | 0,40 |
| *Siphonodictyon coralliphagum* Rützler, 1971 | | 0,60 | 0,63 | 1,31 | 1,94 | 1,77 | 2,06 | 0,51 | 0,57 | 0,66 | 0,86 | 0,80 | 1,06 | 1,11 | 1,63 | 2,80 | 4,91 | 7,20 |
| *Siphonodictyon sp* | | 0,29 | 0,23 | 0,34 | 0,26 | 0,31 | 0,23 | 0,26 | 0,29 | 0,40 | 0,34 | 0,43 | 0,43 | 1,89 | 2,23 | 2,71 | 3,20 | 3,74 |
| *Spirastrella cunctatrix* Schmidt, 1868 | | 0,37 | 0,23 | 0,31 | 0,31 | 0,26 | 0,31 | 0,46 | 0,26 | 0,31 | 0,31 | 0,40 | 0,37 | 0,37 | 0,26 | 0,43 | 0,43 | 0,37 |
| *Spirastrella hartmani* Boury-Esnault, Klautau, Bézac, Wulff & Solé-Cava, 1999 | | 0,20 | 0,14 | 0,17 | 0,40 | 0,20 | 0,23 | 0,23 | 0,26 | 0,23 | 0,43 | 0,26 | 0,43 | 0,31 | 0,43 | 0,51 | 0,40 | 0,43 |
| Total Density (mean ^m-2^) | | 12,7 | 13,0 | 13,9 | 15,4 | 15,6 | 17,3 | 15,8 | 15,5 | 15,5 | 15,2 | 17,1 | 18,7 | 18,0 | 20,0 | 20,7 | 24,4 | 28,4 |
| Total number of species | | 12 | 12 | 12 | 12 | 12 | 12 | 12 | 12 | 12 | 12 | 12 | 12 | 12 | 12 | 12 | 12 | 12 |
|  | |  |  |  |  |  |  |  |  |  |  |  |  |  |  |  |  |  |
| **REEF WALLS** | |  |  |  |  |  |  |  |  |  |  |  |  |  |  |  |  |  |
| *Amphimedon viridis* Duchassaing & Michelotti, 1864 | | 0,23 | 0,17 | 0,17 | 0,23 | 0,17 | 0,34 | 0,29 | 0,23 | 0,20 | 0,20 | 0,23 | 0,26 | 0,31 | 0,26 | 0,23 | 0,20 | 0,17 |
| *Aplysina cauliformis* (Carter, 1882) | | 0,37 | 0,37 | 0,29 | 0,37 | 0,40 | 0,34 | 0,34 | 0,26 | 0,17 | 0,17 | 0,20 | 0,23 | 0,26 | 0,40 | 0,20 | 0,31 | 0,20 |
| *Callyspongia (Cladochalina) tenerrima* Duchassaing & Michelotti, 1864 | | 0,37 | 0,46 | 0,40 | 0,37 | 0,34 | 0,34 | 0,31 | 0,20 | 0,17 | 0,23 | 0,17 | 0,23 | 0,31 | 0,23 | 0,23 | 0,29 | 0,29 |
| *Callyspongia (Cladochalina) vaginalis* (Lamarck, 1814) | | 0,54 | 0,31 | 0,43 | 0,46 | 0,40 | 0,51 | 0,37 | 0,29 | 0,23 | 0,17 | 0,29 | 0,31 | 0,31 | 0,34 | 0,17 | 0,26 | 0,11 |
| *Chondrilla nucula* (Schmidt, 1862) | | 0,34 | 0,31 | 0,31 | 0,26 | 0,40 | 0,37 | 0,26 | 0,26 | 0,17 | 0,20 | 0,20 | 0,23 | 0,29 | 0,40 | 0,17 | 0,26 | 0,20 |
| *Chondrosia sp* | | 0,31 | 0,49 | 0,43 | 0,26 | 0,31 | 0,31 | 0,29 | 0,23 | 0,26 | 0,17 | 0,17 | 0,26 | 0,37 | 0,20 | 0,20 | 0,23 | 0,11 |
| *Cinachyrella alloclada* (Uliczka, 1929) | | 2,09 | 2,03 | 2,26 | 2,17 | 2,14 | 2,11 | 1,89 | 2,71 | 2,80 | 2,63 | 2,29 | 3,31 | 3,40 | 3,17 | 2,80 | 3,43 | 2,83 |
| *Cinachyrella apion* (Uliczka, 1929) | | 1,49 | 1,51 | 1,49 | 1,69 | 1,63 | 1,86 | 2,46 | 2,83 | 2,63 | 2,69 | 3,09 | 3,29 | 2,86 | 2,69 | 2,80 | 2,74 | 3,03 |
| *Cliona celata* (Grant, 1826) | | 2,51 | 2,34 | 2,74 | 2,74 | 3,40 | 3,34 | 4,89 | 4,00 | 3,20 | 3,34 | 3,34 | 3,31 | 4,74 | 4,49 | 4,31 | 4,63 | 4,57 |
| *Cliona varians* (Duchassaing & Michelotti, 1864) | | 0,29 | 0,17 | 0,20 | 0,17 | 0,23 | 0,17 | 0,29 | 0,14 | 0,20 | 0,17 | 0,14 | 0,17 | 0,17 | 0,23 | 0,17 | 0,17 | 0,26 |
| *Desmapsamma anchorata* (Carter, 1882) | | 0,43 | 0,51 | 0,51 | 0,43 | 0,43 | 0,34 | 0,49 | 0,54 | 0,43 | 0,46 | 0,43 | 0,34 | 0,23 | 0,29 | 0,17 | 0,26 | 0,20 |
| *Dysidea etheria* de Laubenfels, 1936 | | 0,43 | 0,43 | 0,46 | 0,31 | 0,34 | 0,51 | 0,40 | 0,40 | 0,31 | 0,34 | 0,26 | 0,20 | 0,17 | 0,17 | 0,17 | 0,26 | 0,26 |
| *Dysidea fragilis* (Montagu, 1818) | | 0,69 | 0,54 | 0,40 | 0,74 | 0,51 | 0,86 | 0,57 | 0,43 | 0,17 | 0,43 | 0,20 | 0,20 | 0,29 | 0,43 | 0,20 | 0,23 | 0,17 |
| *Dysidea sp1* | | 0,66 | 0,40 | 0,49 | 0,51 | 0,63 | 0,91 | 0,43 | 0,34 | 0,31 | 0,37 | 0,17 | 0,34 | 0,23 | 0,29 | 0,14 | 0,17 | 0,23 |
| *Dysidea sp2* | 0,54 | 0,60 | 0,66 | 0,77 | 0,60 | 1,14 | 0,51 | 0,31 | 0,31 | 0,34 | 0,37 | 0,34 | 0,34 | 0,31 | 0,26 | 0,29 | 0,20 |  |
| *Geodia sp1* | 0,23 | 0,29 | 0,23 | 0,23 | 0,26 | 0,40 | 0,23 | 0,29 | 0,20 | 0,23 | 0,26 | 0,34 | 0,40 | 0,23 | 0,14 | 0,14 | 0,14 |  |
| *Geodia sp2* | 0,23 | 0,31 | 0,20 | 0,20 | 0,20 | 0,17 | 0,17 | 0,26 | 0,20 | 0,11 | 0,14 | 0,20 | 0,26 | 0,34 | 0,20 | 0,26 | 0,23 |  |
| *Haliclona (Soestella) caerulea* (Hechtel, 1965) | 0,23 | 0,26 | 0,17 | 0,11 | 0,20 | 0,29 | 0,29 | 0,26 | 0,23 | 0,17 | 0,17 | 0,23 | 0,29 | 0,23 | 0,17 | 0,20 | 0,26 |  |
| *Haliclona implexiformis* (Hechtel,1965) | 0,29 | 0,20 | 0,40 | 0,40 | 0,54 | 0,34 | 0,17 | 0,26 | 0,20 | 0,20 | 0,20 | 0,17 | 0,37 | 0,20 | 0,17 | 0,26 | 0,23 |  |
| *Haliclona sp3* | 0,09 | 0,00 | 0,23 | 0,26 | 0,29 | 0,31 | 0,14 | 0,29 | 0,23 | 0,23 | 0,17 | 0,17 | 0,20 | 0,31 | 0,26 | 0,23 | 0,20 |  |
| *Halicondria sp* | 0,14 | 0,29 | 0,14 | 0,20 | 0,20 | 0,11 | 0,23 | 0,23 | 0,20 | 0,14 | 0,23 | 0,23 | 0,29 | 0,26 | 0,20 | 0,20 | 0,09 |  |
| *Hymeniacidon sp* | 0,29 | 0,29 | 0,14 | 0,37 | 0,23 | 0,31 | 0,29 | 0,20 | 0,11 | 0,20 | 0,17 | 0,23 | 0,29 | 0,43 | 0,14 | 0,17 | 0,20 |  |
| *Ircinia strobilina* (Lamarck, 1816) | 0,26 | 0,23 | 0,37 | 0,37 | 0,20 | 0,49 | 0,17 | 0,11 | 0,17 | 0,29 | 0,14 | 0,23 | 0,29 | 0,20 | 0,17 | 0,31 | 0,11 |  |
| *Laxosuberites sp* | 0,23 | 0,29 | 0,11 | 0,29 | 0,23 | 0,29 | 0,26 | 0,26 | 0,23 | 0,23 | 0,17 | 0,20 | 0,34 | 0,26 | 0,14 | 0,20 | 0,23 |  |
| *Mycale sp2* | 0,31 | 0,37 | 0,37 | 0,29 | 0,37 | 0,34 | 0,17 | 0,43 | 0,17 | 0,26 | 0,17 | 0,23 | 0,31 | 0,37 | 0,20 | 0,14 | 0,23 |  |
| *Mycale sp3* | 0,40 | 0,34 | 0,31 | 0,34 | 0,43 | 0,43 | 0,20 | 0,20 | 0,23 | 0,17 | 0,11 | 0,20 | 0,14 | 0,23 | 0,20 | 0,23 | 0,17 |  |
| *Oscarella sp2* | 0,26 | 0,40 | 0,40 | 0,34 | 0,49 | 0,40 | 0,23 | 0,23 | 0,40 | 0,29 | 0,14 | 0,23 | 0,23 | 0,17 | 0,23 | 0,23 | 0,23 |  |
| *Siphonodictyon coralliphagum* Rützler, 1971 | 0,46 | 0,37 | 0,49 | 0,34 | 0,34 | 0,43 | 0,26 | 0,29 | 0,34 | 0,17 | 0,11 | 0,26 | 1,23 | 2,66 | 3,57 | 3,54 | 4,54 |  |
| *Siphonodictyon sp* | 0,34 | 0,43 | 0,49 | 0,37 | 0,37 | 0,37 | 0,17 | 0,17 | 0,34 | 0,20 | 0,26 | 0,23 | 0,57 | 1,60 | 2,09 | 2,80 | 3,43 |  |
| *Spirastrella cunctatrix* Schmidt, 1868 | 1,31 | 1,46 | 1,51 | 2,06 | 2,03 | 2,06 | 3,97 | 3,20 | 3,23 | 2,83 | 3,14 | 3,69 | 0,91 | 0,20 | 0,20 | 0,17 | 0,29 |  |
| *Spirastrella hartmani* Boury-Esnault, Klautau, Bézac, Wulff & Solé-Cava, 1999 | 0,43 | 0,60 | 0,77 | 0,89 | 0,69 | 0,89 | 0,31 | 0,49 | 0,46 | 0,29 | 0,20 | 0,29 | 0,17 | 0,31 | 0,34 | 0,29 | 0,23 |  |
| *Tedania (Tedania) brasiliensis* Mothes, Hajdu & van Soest, 2000 | 0,34 | 0,40 | 0,29 | 0,49 | 0,29 | 0,54 | 0,63 | 0,40 | 0,26 | 0,37 | 0,37 | 0,23 | 0,29 | 0,29 | 0,23 | 0,29 | 0,23 |  |
| *Tedania (Tedania) ignis* (Duchassaing & Michelotti, 1864) | 0,49 | 0,60 | 0,43 | 0,20 | 0,60 | 0,66 | 0,37 | 0,40 | 0,37 | 0,29 | 0,26 | 0,31 | 0,23 | 0,26 | 0,20 | 0,31 | 0,26 |  |
| *Tethya maza* Selenka, 1879 | 2,77 | 2,86 | 2,89 | 2,83 | 3,17 | 3,31 | 3,09 | 3,31 | 2,77 | 3,57 | 3,09 | 3,69 | 4,40 | 4,06 | 3,37 | 3,40 | 4,06 |  |
| *Tethya rubra* Ribeiro & Muricy, 2004 | 2,69 | 2,60 | 2,74 | 3,11 | 2,77 | 2,94 | 3,66 | 4,37 | 3,97 | 2,94 | 3,63 | 3,37 | 2,97 | 2,86 | 3,66 | 3,74 | 4,00 |  |
| *Vaceletia crypta* (Vacelet, 1977) | 0,40 | 0,43 | 0,37 | 0,34 | 0,20 | 0,31 | 0,37 | 0,37 | 0,40 | 0,23 | 0,40 | 0,31 | 0,26 | 0,23 | 0,17 | 0,26 | 0,17 |  |
| Total Density (mean ^m-2^) | 23,5 | 23,7 | 24,3 | 25,5 | 26,0 | 28,9 | 29,1 | 29,2 | 26,3 | 25,3 | 25,1 | 28,1 | 28,7 | 29,6 | 28,3 | 31,1 | 32,3 |  |
| Total number of species | 36 | 36 | 36 | 36 | 36 | 36 | 36 | 36 | 36 | 36 | 36 | 36 | 36 | 36 | 36 | 36 | 36 |  |
|  |  |  |  |  |  |  |  |  |  |  |  |  |  |  |  |  |  |  |
| **SHALLOW BANKS** |  |  |  |  |  |  |  |  |  |  |  |  |  |  |  |  |  |  |
| *Adocia sp1* | 0,23 | 0,23 | 0,40 | 0,31 | 0,31 | 0,37 | 0,23 | 0,29 | 0,34 | 0,17 | 0,26 | 0,23 | 0,20 | 0,17 | 0,23 | 0,29 | 0,20 |  |
| *Adocia sp2* | 0,14 | 0,26 | 0,20 | 0,14 | 0,23 | 0,17 | 0,20 | 0,26 | 0,26 | 0,26 | 0,14 | 0,26 | 0,26 | 0,23 | 0,20 | 0,20 | 0,20 |  |
| *Amphimedon sp* | 0,11 | 0,20 | 0,29 | 0,29 | 0,14 | 0,11 | 0,14 | 0,20 | 0,23 | 0,26 | 0,20 | 0,17 | 0,17 | 0,11 | 0,20 | 0,17 | 0,14 |  |
| *Amphimedon viridis* Duchassaing & Michelotti, 1864 | 0,11 | 0,17 | 0,14 | 0,20 | 0,26 | 0,20 | 0,23 | 0,20 | 0,23 | 0,20 | 0,26 | 0,17 | 0,23 | 0,20 | 0,23 | 0,20 | 0,17 |  |
| *Aplysina cauliformis* (Carter, 1882) | 0,31 | 0,23 | 0,23 | 0,29 | 0,26 | 0,20 | 0,26 | 0,17 | 0,14 | 0,20 | 0,20 | 0,20 | 0,20 | 0,26 | 0,20 | 0,20 | 0,17 |  |
| *Astroclera sp* | 0,23 | 0,03 | 0,11 | 0,03 | 0,00 | 0,06 | 0,20 | 0,11 | 0,20 | 0,11 | 0,20 | 0,14 | 0,20 | 0,17 | 0,23 | 0,17 | 0,14 |  |
| *Callyspongia (Cladochalina) tenerrima* Duchassaing & Michelotti, 1864 | 0,09 | 0,26 | 0,09 | 0,06 | 0,09 | 0,09 | 0,20 | 0,20 | 0,17 | 0,14 | 0,11 | 0,17 | 0,26 | 0,17 | 0,29 | 0,11 | 0,14 |  |
| *Callyspongia (Cladochalina) vaginalis* (Lamarck, 1814) | 0,26 | 0,20 | 0,11 | 0,26 | 0,11 | 0,17 | 0,17 | 0,17 | 0,17 | 0,14 | 0,14 | 0,17 | 0,23 | 0,23 | 0,20 | 0,11 | 0,14 |  |
| *Chondrilla nucula* (Schmidt, 1862) | 0,17 | 0,23 | 0,34 | 0,26 | 0,14 | 0,31 | 0,20 | 0,17 | 0,23 | 0,14 | 0,17 | 0,20 | 0,20 | 0,23 | 0,20 | 0,14 | 0,20 |  |
| *Chondrosia sp* | 0,14 | 0,06 | 0,14 | 0,09 | 0,20 | 0,17 | 0,23 | 0,20 | 0,20 | 0,14 | 0,14 | 0,17 | 0,17 | 0,14 | 0,11 | 0,11 | 0,09 |  |
| *Cinachyrella alloclada* (Uliczka, 1929) | 1,57 | 1,49 | 1,57 | 1,94 | 2,06 | 1,74 | 1,63 | 1,74 | 2,57 | 2,71 | 2,97 | 4,03 | 3,94 | 4,09 | 3,89 | 3,14 | 3,66 |  |
| *Cinachyrella apion* (Uliczka, 1929) | 1,40 | 1,63 | 1,83 | 2,29 | 2,06 | 1,49 | 2,14 | 1,11 | 2,69 | 3,23 | 3,20 | 4,11 | 3,63 | 3,23 | 3,86 | 3,91 | 5,06 |  |
| *Cinachyrella kuekenthali* (Uliczka, 1929) | 0,06 | 0,29 | 0,17 | 0,00 | 0,09 | 0,09 | 0,23 | 0,49 | 0,26 | 0,20 | 0,26 | 0,20 | 0,29 | 0,20 | 0,26 | 0,23 | 0,20 |  |
| *Cliona celata* (Grant, 1826) | 2,71 | 3,11 | 2,94 | 2,34 | 3,51 | 3,23 | 2,91 | 3,00 | 2,94 | 3,03 | 2,80 | 4,26 | 4,17 | 4,40 | 4,80 | 3,23 | 4,40 |  |
| *Cliona delitrix* Pang, 1973 | 1,31 | 1,69 | 1,37 | 1,14 | 1,23 | 1,23 | 2,00 | 3,11 | 2,51 | 2,89 | 2,94 | 4,14 | 5,17 | 5,03 | 5,20 | 4,69 | 4,71 |  |
| *Cliona varians* (Duchassaing & Michelotti, 1864) | 0,14 | 0,14 | 0,14 | 0,14 | 0,11 | 0,17 | 0,23 | 0,23 | 0,14 | 0,20 | 0,31 | 0,17 | 0,34 | 0,20 | 0,17 | 0,14 | 0,14 |  |
| *Dendrilla sp* | 0,06 | 0,03 | 0,23 | 0,09 | 0,06 | 0,06 | 0,17 | 0,23 | 0,29 | 0,26 | 0,23 | 0,26 | 0,29 | 0,23 | 0,17 | 0,20 | 0,20 |  |
| *Desmapsamma anchorata* (Carter, 1882) | 0,51 | 0,31 | 0,69 | 0,86 | 0,66 | 0,57 | 0,43 | 0,17 | 0,23 | 0,31 | 0,26 | 0,31 | 0,17 | 0,23 | 0,11 | 0,11 | 0,14 |  |
| *Didiscus sp* | 0,40 | 0,37 | 0,43 | 0,54 | 0,37 | 0,34 | 0,20 | 0,31 | 0,20 | 0,23 | 0,20 | 0,26 | 0,26 | 0,29 | 0,20 | 0,17 | 0,20 |  |
| *Dysidea etheria* de Laubenfels, 1936 | 0,51 | 0,46 | 0,51 | 0,31 | 0,74 | 0,29 | 0,34 | 0,20 | 0,26 | 0,23 | 0,23 | 0,20 | 0,14 | 0,23 | 0,17 | 0,26 | 0,11 |  |
| *Dysidea fragilis* (Montagu, 1818) | 0,23 | 0,49 | 0,71 | 0,31 | 0,51 | 0,37 | 0,20 | 0,29 | 0,40 | 0,20 | 0,17 | 0,29 | 0,31 | 0,17 | 0,17 | 0,11 | 0,11 |  |
| *Dysidea robusta* Vilanova & Muricy, 2001 | 0,60 | 0,46 | 0,51 | 0,43 | 0,46 | 0,60 | 0,31 | 0,37 | 0,26 | 0,29 | 0,14 | 0,26 | 0,17 | 0,17 | 0,17 | 0,11 | 0,14 |  |
| *Dysidea sp1* | 0,40 | 0,37 | 0,51 | 0,46 | 0,31 | 0,60 | 0,31 | 0,31 | 0,31 | 0,23 | 0,23 | 0,26 | 0,20 | 0,23 | 0,17 | 0,20 | 0,20 |  |
| *Dysidea sp2* | 0,09 | 0,11 | 0,17 | 0,03 | 0,20 | 0,29 | 0,26 | 0,20 | 0,20 | 0,17 | 0,23 | 0,23 | 0,23 | 0,23 | 0,29 | 0,23 | 0,17 |  |
| *Dysidea variabilis* (Duchassaing & Michelotti, 1864) | 0,20 | 0,69 | 0,40 | 0,54 | 0,40 | 0,49 | 0,23 | 0,40 | 0,20 | 0,20 | 0,23 | 0,20 | 0,20 | 0,20 | 0,17 | 0,17 | 0,14 |  |
| *Erylus formosus* Sollas, 1886 | 0,09 | 0,23 | 0,11 | 0,23 | 0,03 | 0,09 | 0,14 | 0,26 | 0,14 | 0,11 | 0,29 | 0,20 | 0,20 | 0,20 | 0,29 | 0,14 | 0,11 |  |
| *Gastrophanella stylifera* Mothes & Silva, 1999 | 0,06 | 0,26 | 0,31 | 0,23 | 0,06 | 0,06 | 0,23 | 0,23 | 0,20 | 0,20 | 0,26 | 0,23 | 0,20 | 0,20 | 0,17 | 0,06 | 0,26 |  |
| *Geodia corticostylifera* Hajdu, Muricy, Custodio, Russo & Peixinho, 1992 | 0,06 | 0,09 | 0,23 | 0,17 | 0,11 | 0,11 | 0,17 | 0,20 | 0,14 | 0,17 | 0,14 | 0,23 | 0,20 | 0,17 | 0,11 | 0,14 | 0,20 |  |
| *Geodia gibberosa* Lamarck, 1815 | 0,00 | 0,11 | 0,06 | 0,20 | 0,14 | 0,11 | 0,14 | 0,11 | 0,14 | 0,14 | 0,20 | 0,20 | 0,23 | 0,17 | 0,20 | 0,11 | 0,20 |  |
| *Geodia papyracea* Hechtel, 1965 | 0,14 | 0,17 | 0,11 | 0,23 | 0,11 | 0,26 | 0,23 | 0,20 | 0,11 | 0,14 | 0,20 | 0,20 | 0,17 | 0,14 | 0,26 | 0,14 | 0,17 |  |
| *Haliclona caerulea* (Hechtel, 1965) | 0,17 | 0,34 | 0,63 | 0,14 | 0,23 | 0,66 | 0,17 | 0,23 | 0,29 | 0,11 | 0,14 | 0,20 | 0,14 | 0,17 | 0,17 | 0,14 | 0,17 |  |
| *Haliclona implexiformis* (Hechtel,1965) | 0,09 | 0,31 | 0,43 | 0,29 | 0,34 | 0,17 | 0,23 | 0,26 | 0,23 | 0,20 | 0,14 | 0,14 | 0,23 | 0,17 | 0,17 | 0,11 | 0,11 |  |
| *Haliclona sp1* | 0,00 | 0,31 | 0,29 | 0,26 | 0,34 | 0,03 | 0,11 | 0,23 | 0,14 | 0,20 | 0,17 | 0,20 | 0,23 | 0,29 | 0,11 | 0,17 | 0,17 |  |
| *Haliclona sp2* | 0,29 | 0,23 | 0,43 | 0,37 | 0,20 | 0,26 | 0,23 | 0,20 | 0,29 | 0,26 | 0,23 | 0,14 | 0,17 | 0,14 | 0,17 | 0,11 | 0,17 |  |
| *Haliclona sp3* | 1,11 | 0,94 | 1,17 | 2,03 | 0,97 | 1,31 | 1,89 | 1,46 | 2,00 | 1,91 | 2,46 | 3,26 | 1,74 | 1,69 | 1,43 | 1,20 | 1,29 |  |
| *Haliclona sp4* | 0,23 | 0,06 | 0,14 | 0,20 | 0,20 | 0,06 | 0,26 | 0,23 | 0,23 | 0,23 | 0,20 | 0,31 | 0,26 | 0,23 | 0,23 | 0,29 | 0,14 |  |
| *Halicondria sp* | 0,54 | 0,14 | 0,34 | 0,46 | 0,23 | 0,23 | 0,43 | 0,26 | 0,23 | 0,40 | 0,20 | 0,23 | 0,29 | 0,11 | 0,26 | 0,11 | 0,17 |  |
| *Hymeniacidon perlevis* (Montagu, 1818) | 0,26 | 0,31 | 0,20 | 0,14 | 0,29 | 0,26 | 0,26 | 0,31 | 0,29 | 0,26 | 0,20 | 0,23 | 0,20 | 0,29 | 0,20 | 0,23 | 0,20 |  |
| *Ircinia strobilina* (Lamarck, 1816) | 0,37 | 0,49 | 0,51 | 0,74 | 0,66 | 0,69 | 0,37 | 0,40 | 0,23 | 0,31 | 0,31 | 0,23 | 0,17 | 0,20 | 0,23 | 0,09 | 0,11 |  |
| *Jaspis sp* | 0,09 | 0,00 | 0,23 | 0,11 | 0,26 | 0,11 | 0,14 | 0,20 | 0,26 | 0,20 | 0,23 | 0,23 | 0,29 | 0,23 | 0,17 | 0,14 | 0,23 |  |
| *Laxosuberites sp* | 0,69 | 0,11 | 0,34 | 0,34 | 0,37 | 0,37 | 0,37 | 0,20 | 0,23 | 0,14 | 0,23 | 0,20 | 0,23 | 0,17 | 0,20 | 0,20 | 0,17 |  |
| *Mycale laevis* (Carter, 1882) | 0,20 | 0,14 | 0,23 | 0,14 | 0,11 | 0,17 | 0,26 | 0,29 | 0,20 | 0,14 | 0,26 | 0,31 | 0,29 | 0,17 | 0,20 | 0,17 | 0,20 |  |
| *Mycale laxissima* (Duchassaing & Michelotti, 1864) | 0,34 | 0,46 | 0,43 | 0,51 | 0,20 | 0,57 | 0,29 | 0,31 | 0,17 | 0,17 | 0,17 | 0,29 | 0,29 | 0,17 | 0,14 | 0,20 | 0,11 |  |
| *Mycale microsigmatosa* Arndt, 1927 | 0,06 | 0,26 | 0,17 | 0,26 | 0,20 | 0,09 | 0,26 | 0,23 | 0,23 | 0,14 | 0,14 | 0,20 | 0,20 | 0,29 | 0,14 | 0,17 | 0,14 |  |
| *Myxilla sp* | 0,06 | 0,11 | 0,26 | 0,34 | 0,29 | 0,34 | 0,17 | 0,20 | 0,14 | 0,17 | 0,17 | 0,26 | 0,23 | 0,17 | 0,11 | 0,20 | 0,14 |  |
| *Oscarella sp1* | 0,03 | 0,03 | 0,09 | 0,09 | 0,06 | 0,03 | 0,20 | 0,14 | 0,14 | 0,17 | 0,17 | 0,20 | 0,11 | 0,14 | 0,20 | 0,20 | 0,17 |  |
| *Oscarella sp2* | 0,03 | 0,03 | 0,06 | 0,06 | 0,09 | 0,06 | 0,14 | 0,14 | 0,11 | 0,11 | 0,14 | 0,20 | 0,17 | 0,17 | 0,11 | 0,11 | 0,26 |  |
| *Petromica ciocalyptoides* (van Soest & Zea, 1986) | 0,00 | 0,09 | 0,00 | 0,00 | 0,00 | 0,03 | 0,20 | 0,17 | 0,11 | 0,11 | 0,17 | 0,23 | 0,17 | 0,17 | 0,20 | 0,11 | 0,20 |  |
| *Siphonodictyon coralliphagum* Rützler, 1971 | 0,77 | 0,43 | 0,66 | 0,77 | 0,77 | 0,57 | 0,66 | 0,20 | 0,26 | 0,31 | 0,20 | 0,26 | 0,80 | 0,43 | 0,69 | 0,89 | 0,80 |  |
| *Siphonodictyon sp* | 0,23 | 0,06 | 0,23 | 0,43 | 0,63 | 0,54 | 0,23 | 0,20 | 0,17 | 0,23 | 0,23 | 0,26 | 0,97 | 0,74 | 0,97 | 0,80 | 0,54 |  |
| *Spirastrella cunctatrix* Schmidt, 1868 | 0,34 | 0,60 | 0,43 | 0,26 | 0,31 | 0,23 | 0,23 | 0,29 | 0,31 | 0,17 | 0,17 | 0,26 | 0,23 | 0,20 | 0,11 | 0,14 | 0,14 |  |
| *Spirastrella hartmani* Boury-Esnault, Klautau, Bézac, Wulff & Solé-Cava, 1999 | 0,23 | 0,20 | 0,29 | 0,17 | 0,11 | 0,20 | 0,20 | 0,17 | 0,23 | 0,17 | 0,20 | 0,23 | 0,29 | 0,20 | 0,23 | 0,20 | 0,26 |  |
| *Suberites aurantiacus* (Duchassaing & Michelotti, 1864) | 0,40 | 0,20 | 0,26 | 0,23 | 0,49 | 0,31 | 0,29 | 0,23 | 0,17 | 0,26 | 0,29 | 0,23 | 0,29 | 0,23 | 0,29 | 0,20 | 0,34 |  |
| *Tedania (Tedania) brasiliensis* Mothes, Hajdu & van Soest, 2000 | 0,40 | 0,20 | 0,34 | 0,17 | 0,51 | 0,06 | 0,34 | 0,14 | 0,23 | 0,23 | 0,31 | 0,23 | 0,17 | 0,20 | 0,20 | 0,11 | 0,14 |  |
| *Tedania (Tedania) ignis* (Duchassaing & Michelotti, 1864) | 0,09 | 0,40 | 0,00 | 0,31 | 0,29 | 0,26 | 0,23 | 0,37 | 0,23 | 0,17 | 0,29 | 0,23 | 0,37 | 0,26 | 0,14 | 0,14 | 0,37 |  |
| *Terpios sp1* | 0,06 | 0,29 | 0,46 | 0,06 | 0,23 | 0,00 | 0,20 | 0,20 | 0,23 | 0,20 | 0,20 | 0,17 | 0,29 | 0,17 | 0,20 | 0,14 | 0,20 |  |
| *Terpios sp2* | 0,09 | 0,17 | 0,09 | 0,20 | 0,11 | 0,06 | 0,20 | 0,20 | 0,17 | 0,14 | 0,17 | 0,23 | 0,17 | 0,17 | 0,17 | 0,20 | 0,17 |  |
| *Tethya maza* Selenka, 1879 | 1,46 | 1,37 | 1,63 | 1,97 | 1,66 | 1,71 | 1,43 | 1,23 | 1,49 | 1,83 | 1,51 | 2,77 | 2,03 | 1,57 | 2,29 | 2,40 | 1,11 |  |
| *Tethya rubra* Ribeiro & Muricy, 2004 | 1,74 | 1,40 | 1,31 | 1,60 | 1,66 | 1,57 | 2,57 | 3,11 | 3,06 | 2,74 | 2,57 | 3,09 | 2,46 | 2,09 | 2,69 | 2,91 | 1,46 |  |
| *Tribachion schimidtii* Weltner, 1882 | 0,63 | 0,29 | 0,34 | 0,51 | 0,49 | 0,40 | 0,49 | 0,31 | 0,23 | 0,31 | 0,31 | 0,29 | 0,23 | 0,23 | 0,23 | 0,29 | 0,20 |  |
| *Vaceletia crypta* (Vacelet, 1977) | 0,09 | 0,34 | 0,26 | 0,03 | 0,14 | 0,23 | 0,26 | 0,34 | 0,14 | 0,17 | 0,29 | 0,31 | 0,11 | 0,17 | 0,20 | 0,20 | 0,14 |  |
| *Vosmaeria sp* | 0,06 | 0,09 | 0,00 | 0,06 | 0,11 | 0,09 | 0,14 | 0,20 | 0,17 | 0,17 | 0,11 | 0,31 | 0,17 | 0,20 | 0,23 | 0,14 | 0,17 |  |
| *Vulcanella sp* | 0,17 | 0,00 | 0,40 | 0,11 | 0,23 | 0,20 | 0,23 | 0,14 | 0,17 | 0,23 | 0,26 | 0,23 | 0,20 | 0,14 | 0,11 | 0,11 | 0,20 |  |
| Total Density (mean ^m-2^) | 23,6 | 24,7 | 27,4 | 27,8 | 27,7 | 25,9 | 28,2 | 27,9 | 29,1 | 29,5 | 30,2 | 38,3 | 36,8 | 33,9 | 36,1 | 32,1 | 32,6 |  |
| Total number of species | 60 | 62 | 60 | 61 | 61 | 63 | 63 | 63 | 63 | 63 | 63 | 63 | 63 | 63 | 63 | 63 | 63 |  |
