# Supplementary material for: Tolerance of Sponge Assemblages to Temperature Anomalies: Resilience and Proliferation of Sponges following the 1997–8 El-Niño Southern Oscillation
Source: PLoS One. 2013 Oct 7;8(10):e76441. doi: 10.1371/journal.pone.0076441 (PMC3792017; doi:10.1371/journal.pone.0076441)
Supplement: Table S2 — Statistical differences in sponge assemblages between Reefs/Years measured from 1995 to 2011 tested by a distance-based permutational multivariate analysis of variance, PERMANOVA. (DOCX) [file pone.0076441.s002.docx]

**Table S2** Statistical differences in sponge assemblages between Reefs/Years measured from 1995 to 2011 tested by a distance-based permutational multivariate analysis of variance, PERMANOVA.

**ERT**

| Source | df | SS | MS | Pseudo-F | P(perm) | Unique perms |
| --- | --- | --- | --- | --- | --- | --- |
| Reefs | 3 | 566,35 | 188,78 | 1,1066 | 0,3234 | 4976 |
| Years (Reefs) | 8 | 2833,8 | 354,22 | 2,0764 | 0,0004 | 4957 |
| Res | 56 | 9553,2 | 170,59 |  |  |  |
| Total | 67 | 12867 |  |  |  |  |

**CRW**

| Source | df | SS | MS | Pseudo-F | P(perm) | Unique perms |
| --- | --- | --- | --- | --- | --- | --- |
| Reefs | 3 | 210,08 | 70,027 | 0,53757 | 0,863 | 4986 |
| Years (Reefs) | 8 | 1232,9 | 154,12 | 1,8244 | 0,0208 | 4967 |
| Res | 56 | 4730,6 | 84,476 |  |  |  |
| Total | 67 | 6228,9 |  |  |  |  |

**SBR**

| Source | df | SS | MS | Pseudo-F | P(perm) | Unique perms |
| --- | --- | --- | --- | --- | --- | --- |
| Reefs | 3 | 1232,7 | 410,89 | 1,7857 | 0,0146 | 4985 |
| Years (Reefs) | 8 | 5972,9 | 746,62 | 3,2447 | 0,0002 | 4949 |
| Res | 56 | 12886 | 230,1 |  |  |  |
| Total | 67 | 19654 |  |  |  |  |
